# Uncovering phenotypic heterogeneity through research autopsy in lethal prostate cancer

**DOI:** 10.1172/JCI195102

**Published:** 2025-08-01

**Authors:** Sylvie S.W. Chan, Osvaldas Vainauskas, Gerhardt Attard

**Affiliations:** Department of Oncology, University College London Cancer Institute, University College London, London, United Kingdom.

## Abstract

Tumor heterogeneity in metastatic prostate cancer (mPC) is well established, but comprehensive characterization using routine sampling remains challenging. Autopsy-based research addresses this obstacle by enabling broad tissue collection within individual patients after treatment. In this issue of the *JCI*, Roudier et al. analyzed samples from a mPC research autopsy cohort, revealing extensive inter- and intratumor heterogeneity across patients and at the cellular level. The authors associated this variability with genomic, phenotypic, and clinical features and explored the importance of tumors expressing both androgen receptor and neuroendocrine markers. Their findings demonstrate heterogeneity across metastatic sites that may influence treatment response and clinical outcomes, informing future therapeutic strategies in mPC.

## Heterogeneity of prostate cancer

Prostate cancer (PC) is among the most commonly diagnosed cancers in men, but despite recent advances in treatment, metastatic disease often remains lethal (1). A deeper understanding of the biological mechanisms driving treatment resistance in PC is critical to enable ongoing therapeutic development and improve patient outcomes.

PC growth is initially driven by androgens. Treatment with androgen deprivation and/or androgen receptor (AR) pathway inhibition is usually highly successful. However, resistance inevitably emerges and is frequently driven by *AR* gene alterations. These include copy number changes, point mutations, and structural variants, any of which can hypersensitize AR to castrate levels of androgens, or expand ligand specificity to nonandrogen steroids, maintaining AR activity despite therapy (2).

Transformation into neuroendocrine PC (NEPC) from prostate adenocarcinoma is also increasingly recognized, reported in up to 20% of late-stage PC cases (3, 4). NEPC is characterized by expression of neuroendocrine (NE) markers, androgen-independent growth, and low-serum prostate-specific antigen (PSA) production relative to disease volume, which limits the utility of PSA as a disease biomarker (5). Both epigenetic changes, such as DNA methylation, and specific genomic events are associated with lineage plasticity and the transition to a NE state (6–8). Clinically, NEPC is often defined by visceral metastases and rapidly proliferating tumors. It is associated with very poor prognosis and remains a diagnostic and therapeutic challenge (5, 9).

Additional PC subtypes have been identified but remain poorly understood; for instance, the amphicrine phenotype is characterized by positivity of both AR and NE immunohistochemical (IHC) markers, while double-negative disease displays neither (10). Because these subtypes usually emerge in the context of response to hormone therapies, androgen deprivation therapy is continued, and additional treatment is considered. For NEPC, platinum-based chemotherapy is commonly added, mirroring treatment strategies for NE disease of other primary cancers (6).

There is an urgent need to better characterize AR-independent driver events and understand interactions between AR^+^ and AR^–^ cell populations, particularly as their coexistence could increase the complexity of providing effective treatment.

## Investigating PC heterogeneity using research autopsies

Research autopsies offer the opportunity to comprehensively sample primary and metastatic lesions, including those undetected by clinical imaging. This level of collection surpasses routine sampling (11), as it enables high-resolution spatial, inter-, and intratumor characterization, essential for understanding tumor heterogeneity. Maintaining tissue quality requires a rapid-response team, while recruitment is influenced by cultural and societal attitudes surrounding postmortem examination (12).

Although research autopsies allow thorough tissue collection, samples are only informative of end-stage disease and do not capture dynamic processes such as specific treatment-induced changes. Furthermore, as treatment paradigms change, the applicability of findings made from postmortem samples may become less relevant to contemporary standards of care.

Several landmark studies have investigated PC heterogeneity via postmortem analysis (13–17), offering insights into driver events, disease phylogenetics, metastatic dissemination, and, ultimately, treatment resistance. These studies have demonstrated that, while key driver events are often shared across all metastases, clonal diversity is also common, reflecting a complex and branching evolutionary process (13). Differential treatment responses between metastatic sites — for example, response in bone sites but progression in liver lesions — highlight clinical heterogeneity (18), but the interplay with clonal architecture remains poorly understood.

## Linking molecular heterogeneity to clinical features

**Figure 1:**
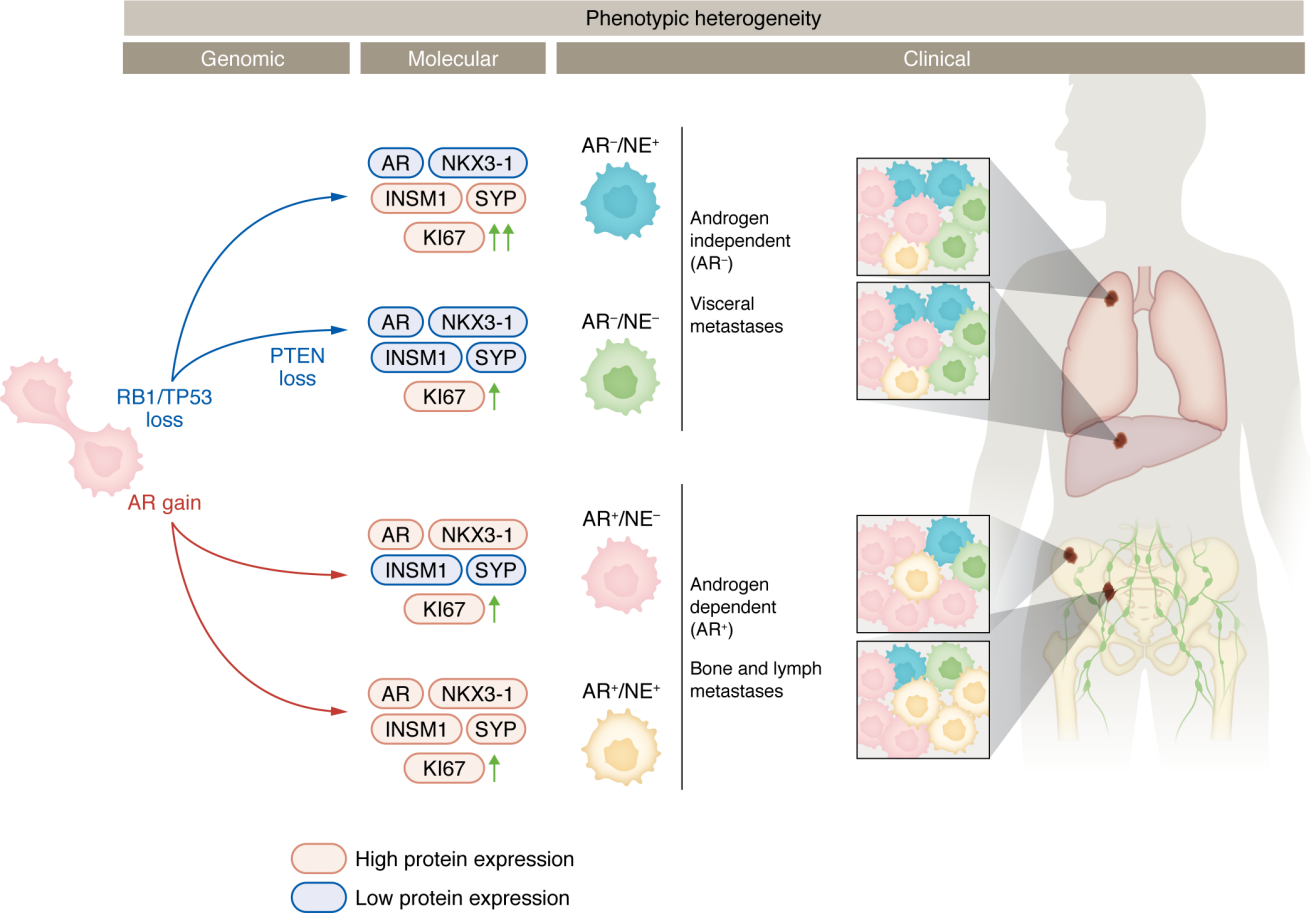
Metastatic PC shows phenotypic heterogeneity. Treated metastatic PC cells show genomic gains and/or losses that associate with molecular changes and result in phenotypic differentiation. The four phenotypic categories — AR^+^/NE^–^, AR^–^/NE^–^, AR^–^/NE^+^, and AR^+^/NE^+^ — are associated with differences in androgen dependence, proliferative capacity (as seen by Ki-67 expression), and metastatic locations.

In this issue of the *JCI*, Roudier et al. analyzed 637 tumor samples from 52 patients in the University of Washington’s Prostate Cancer Rapid Autopsy Program to investigate phenotypic heterogeneity in metastatic PC (mPC) (19). Tumors were classified into four molecular subtypes (AR^+^/NE^–^, AR^–^/NE^+^, AR^+^/NE^+,^ and AR^–^/NE^–^) based on AR signaling and NE IHC markers (20). This categorization allowed standardized assessment for the coexistence of androgen-expressing and NE cells across many samples. As expected, AR^+^ tumors were most commonly seen, but 71% of patients exhibited multiple subtypes across metastatic sites. Notably, AR^–^ disease was also widely identified, particularly in visceral metastases (19) (Figure 1).

Genomic profiling revealed subtype-specific patterns: *AR* amplification was enriched in AR^+^ tumors, *RB1* and *TP53* biallelic loss were common in AR^–^ tumors, and *PTEN* alterations were predominantly seen in AR^–^/NE^–^ lesions (Figure 1). Additionally, the Ki-67 proliferative index was highest in AR^–^/NE^+^ tumors, also correlating with biallelic *RB1* loss and the absence of *AR* alterations (Figure 1). However, this same genomic pattern was also detected across other subtypes, suggesting that transitions between phenotypes also have nongenomic drivers and that there may be a continuum of subtypes across which molecular changes overlap (19).

## Understanding intrametastatic heterogeneity

To further explore phenotypic admixture, the authors analyzed an independent single-cell RNA-sequencing dataset using multigene signatures. Intriguingly, AR^+^/NE^–^ tumors often contained AR^–^/NE^–^ cells, while AR^–^ tumors were more homogeneous, in keeping with the IHC findings from the Rapid Autopsy Program. These findings raise biologically and clinically interesting possibilities, such as intermediate states of AR independence, loss of AR signaling without adoption of the NE phenotype, or traditional NE disease gaining AR positivity.

Roudier et al. (19) also improved understanding of the AR^+^/NE^+^ amphicrine subtype (10). They elucidated two distinct patterns — true amphicrine and mixed/biphenotypic — the former group coexpressing AR and NE markers while being molecularly similar to AR^+^/NE^–^ tumors and the latter an admixture of AR^+^/NE^–^ and AR^–^/NE^+^ cell populations. By further interrogating a biphenotypic case using single-nucleus RNA sequencing (snRNA-seq) and single-nucleus assay for transposase-accessible chromatin sequencing (snATAC-seq), they showed that different transcriptional patterns were linked to underlying chromatin and copy number changes, underscoring a multilayered basis for phenotypic diversity (19).

Another case displayed metastases with multiple distinct histomorphologies and molecular subtypes while harboring common driver alterations, including *RB1* and *PTEN* loss (19). snRNA-seq analysis found corresponding cell populations within a primary tumor specimen, while snATAC-seq revealed four molecular clusters — however, all clusters shared copy number variants in core driver alterations that also overlapped with bulk genomic findings from the primary tumor. This detailed analysis provides strong evidence that tumors arise from a common clonal origin but also diversify epigenetically and phenotypically, which confirms the merit in utilizing shared driver alterations as therapeutic targets.

## Conclusions and future directions

Establishing such an extensive postmortem program is a notable achievement, and we acknowledge the profound contributions of participants and their loved ones, alongside the work of the investigator team. Through their analysis, Roudier et al. provide compelling evidence that phenotypic and molecular heterogeneity in mPC is pervasive, complex, and potentially clinically important (19). In this study, AR^–^ tumors were surprisingly common and showed low molecular heterogeneity, both within individual patients and across metastatic sites. These tumors were predominantly found in visceral metastases and were enriched for *RB1*, *TP53*, and *PTEN* loss (19) — alterations consistently associated with poor prognosis in mPC that have been proposed as biomarkers for treatment with cabazitaxel plus carboplatin (21).

The coexistence of different phenotypes may have major therapeutic implications, given their distinctly different expression of cell surface markers and molecular drivers (9). Eradication may require combinations of therapies that may not be safe or could be accompanied by severe toxicity. The best solution may be to prevent cell differentiation through early detection and targeted treatment. The first step to achieving this goal is to obtain a clearer understanding of the molecular underpinnings of these molecular subtypes and temporally resolve clone dynamics to better understand how treatment pressures trigger their emergence. Additionally, Roudier et al. highlight some of the limitations of a single biopsy, which is often performed to detect NEPC and guide treatment (19). Due to phenotypic variability, a single biopsy may only reflect a fraction of the extent of phenotypes existing across metastatic sites. Since multiple tissue biopsies are impractical, noninvasive diagnostic methods such as liquid biopsy (22) or functional imaging for NEPC (23) could be options.

Ultimately, Roudier et al. have convincingly demonstrated that multiple molecular subtypes commonly coexist at both inter- and intratumor levels (19). The authors have provided insights into the amphicrine subtype by utilizing high-resolution cellular techniques. Their multimodal study highlights how metastatic lesions can diverge in morphology, proliferative capacity, and signaling states despite sharing common clonal origins. Although associations with clinical outcomes were not identified — likely due to small sample size and retrospective analysis — their findings raise critical questions about the interplay between tumor heterogeneity, genomic background, AR^–^ phenotypes, and metastatic potential (19).

## Version 1 08/01/2025

Electronic publication
